# Identification of PANoptosis-related biomarkers and analysis of prognostic values in head and neck squamous cell carcinoma

**DOI:** 10.1038/s41598-024-60441-8

**Published:** 2024-04-29

**Authors:** Ping Yang, Guangzhao Huang, Yulin Li, Lang Yu, Zili Yin, Qian Li

**Affiliations:** 1 Department of Anesthesiology, West China Hospital, Sichuan University, Chengdu, 610041 China; 2 Department of Anesthesiology, Chongqing University Three Gorges Hospital, Chongqing, 404100 China; 3 Division of Oral Ecology and Biochemistry, Tohoku University Graduate School of Dentistry, Sendai, 980-8575 Japan; 4 Department of stomatology, Zigong Third People’s Hospital, Zigong, 643020 China; 5 Department of Stomatology, Yunyang County People’s Hospital, Chongqing, 404500 China; 6 Department of Anesthesiology, West China Hospital, Sichuan University, 37 Guo Xue Alley, Wuhou District, Chengdu, Sichuan China

**Keywords:** PANoptosis, Immunotherapy, HNSCC, *FADD*, *ZBP1*, Cancer, Biomarkers

## Abstract

PANoptosis plays a crucial role in cancer initiation and progression. However, the roles of PANoptosis-related genes (PARGs) in the prognosis and immune landscape of head and neck squamous cell carcinoma (HNSCC) remain unclear. Integrated bioinformatics analyses based on the data of HNSCC patients in the TCGA database were conducted. We extracted 48 PARGs expression profile and then conducted differentially expressed analysis, following building a Cox model to predict the survival of HNSCC patients. Subsequently, the relationships between the risk score, immune landscape, chemo-, and immune-therapy responses were analyzed, respectively. Moreover, we investigated the prognostic value, and further predicted the pathways influenced by PARGs. Finally, we identified the biological function of crucial PARGs. A total of 18 differentially expressed PARGs were identified in HNSCC, and a Cox model including *CASP8*, *FADD*, *NLRP1*, *TNF*, and *ZBP1* was constructed, which showed that the risk score was associated with the prognosis as well as immune infiltration of HNSCC patients, and the risk score could be regarded as an independent biomarker. Additionally, patients with high-risk score might be an indicator of lymph node metastasis and advanced clinical stage. High-risk scores also contributed to the chemotherapy resistance and immune escape of HNSCC patients. In addition, *FADD* and *ZBP1* played a crucial role in various cancer-related pathways, such as the MAPK, WNT, and MTOR signaling pathways. On the other hand, we suggested that *FADD* facilitated the progression and 5-fluorouracil (5-FU) resistance of HNSCC cells. A signature based on PANoptosis showed great predictive power for lymph node metastasis and advanced stage, suggesting that the risk score might be an independent prognostic biomarker for HNSCC. Meanwhile, *FADD*, identified as a prognostic biomarker, may represent an effective therapeutic target for HNSCC.

## Introduction

Head and neck squamous cell carcinoma (HNSCC) is the seventh most common cancer type worldwide, with more than 850,000 new cases and 400,000 deaths per year^1^. HNSCC is characterized by chemoresistance, recurrence, lymph node and distant metastasis, which lead to an unsatisfactory prognosis^2^. Despite advances in therapeutic strategies, the 5-year overall survival rate is also unsatisfying^3^, being lower than 65% and lower than 40% in advanced stage cases^4,5^. Therefore, it is urgent to clarify the mechanism of HNSCC progression and thereby identify effective therapeutic targets.

As a widespread phenomenon in organisms, cell death is crucial for maintaining homeostasis. The imbalance between death and growth is the direct reason for tumorigenesis^6^. Programmed cell death (PCD), including pyroptosis, apoptosis, and necroptosis, is the mode of active cell death determined by genetic information. Recently, PANoptosis was identified as a new PCD pathway^7^, which is defined as an inflammatory PCD pathway regulated by the PANoptosome complex. PANoptosis contains the vital molecules and key features of traditional PCD pathways, which can crosstalk and influence their mutual relationship^8^, while the typical features of PANoptosis cannot be accounted for by any of these PCD pathways alone. PANoptosis has been reported to play a crucial role in tumor initiation and progression. For instance, the PANoptosis-Related lncRNA SNHG7 is involved in chemoresistance and metastasis in colon adenocarcinoma^9^. The characterization of PANoptosis patterns can also provide a roadmap for predicting survival and immunotherapy response^10^. Recently, the majority of studies have focused on PANoptosis to identify candidate biomarkers for various cancers^11,12^. For instance, PANoptosis-relevant subgroups could evaluate the prognosis and immune landscape of liver hepatocellular carcinoma (LIHC)^11^, suggesting that understanding PANoptosis might provide strategies for the clinical therapy of LIHC. However, few studies have focused on the contribution of PANoptosis to HNSCC prognostic value and therapy resistance. Recently. Feng Gao et al. indicated that the PANoptosis pattern could predict the prognosis and immunotherapy response of HNSCC patients^13^. Targeting PANoptosis might facilitate the development of more effective treatment approaches for HNSCC. Therefore, the discovery of relevant biomarkers of PANoptosis in HNSCC is crucial. A better understanding of the role of PANoptosis in HNSCC might be helpful to provide new insights into diagnosis and targeted therapy.

In this study, prognostic PANoptosis-related genes (PARGs) in HNSCC patients were identified by analyzing datasets obtained from The cancer genome atlas (TCGA). Furthermore, we investigated the prognostic value of PANoptosis by building a risk model, and the predictive power of the risk model was validated. Moreover, alterations in the apoptotic program are associated with the cancer response to drugs^14^. Hence, we explored the correlation between PANoptosis-related biomarkers and drug susceptibility. On the other hand, we analyzed the role of PANoptosis in immune infiltration and the immune response. Subsequently, we predicted the potential mechanism of PANoptosis-related biomarkers via gene set enrichment analysis (GSEA). Finally, we investigated the biological function of PANoptosis-related biomarkers. Taken together, this research may provide new insights into the diagnosis and targeted therapy of HNSCC.

## Material and methods

### Transcriptome data preparation

The transcriptome profile and relevant clinical information of HNSCC patients were downloaded from TCGA (https://portal.gdc.cancer.gov/) database. Cases without survival data were excluded. A total of 44 normal controls and 519 tumor cases were obtained. 48 PARGs were obtained from previous studies (Table S1)^11,15–17^, following the selection of differentially expressed PARGs (DEPARGs) with the cut-off criteria |log_2_ (FoldChange (FC))|> 1 and p-value < 0.05.

### To identify the prognostic value of PARGs in HNSCC

According to the identified DEPARGs, we merged the gene expression level and survival data of each patient. Furthermore, the selected genes were enrolled in multivariate and stepwise Cox regression analyses to construct a risk model. In accordance with the risk formula, we calculated the risk score of each patient according to risk score model, and divided patients into high- and low-risk groups based on the risk score median. Kaplan–Meier curves along with long rank tests were used to identify the predictive power of risk model. Moreover, we used univariate and multivariate Cox regression analyses to explore whether the risk score could be regarded as an independent prognostic biomarker for HNSCC. The confidence interval was given with HR. Meanwhile, we identified the prognostic value of PARGs enrolled in risk model.

### Tumor microenvironment (TME) analysis

According to genome expression, we calculated the immune cell levels via single sample gene set enrichment analysis (ssGSEA) in the R software “CIBERSORT” package^18,19^. We further explored the correlation between prognosis-related PARGs and immune cell levels. In addition, we predicted the immune cell and function scores of HNSCC patients in the R software packages “GVSA” and “GSEAbase”, and then investigated the difference in immune infiltration between the high- and low-risk groups. Meanwhile, we downloaded the stemness score based on DNA methylation (DNAss) and RNA (RNAss) from UCSC Xena (http://xena.ucsc.edu/), following exploration of the correlation between stemness score and PARGs.

### Immunotherapy and drug therapy analysis

Tumor mutation burden (TMB) is regarded as a predictor of immunotherapeutic response. We obtained HNSCC patients’ TMB data from the UCSC Xena database (https://xena.ucsc.edu/) and further explored the correlation between TMB and prognosis-related PARGs. Furthermore, we explored the co-expression of PARGs and immune checkpoints (ICPs). We also downloaded the immunotherapy data of HNSCC patients from the TCIA database (https://tcia.at/home) and investigated the role of PARGs in HNSCC immunotherapy. Moreover, we obtained drug susceptibility data from the CellMiner database (https://discover.nci.nih.gov/cellminer/home.do) and further investigated the role of PANoptosis in drug resistance.

### Gene set enrichment analysis (GSEA)

GSEA was used to predict the KEGG pathways affected by PANoptosis. Generally, prognosis-related PARGs were divided into high- and low-expression groups in accordance with the expression median. Subsequently, the genome expression file and group file were imported into GSEA software to screen KEGG pathways influenced by PARGs. The top 5 KEGG pathways were listed.

### Cell culture and transfection

HNSCC cell lines HN12, SCC9, SCC15, Cal27, and human oral keratinocyte (HOK) were cultured in Dulbecco’s modified Eagle’s medium (DMEM) with 10% fetal bovine serum (FBS) and 1% penicillin–streptomycin at 37 °C with 5% CO_2_. All cell lines were obtained from ATCC. Small interfering RNAs (siRNAs) targeting Fas associated via death domain (*FADD*) were designed and synthesized. Cells were seeded in 6-well plates, and when the density reached 80%, siRNAs were transfected following the instructions of the Lipofectamine 3000 Kit. Total mRNAs of transfected cells were collected for analysis after 2 days. The siRNA sequences were as follows: Sense-GAGUCACUGAGAAUCUGGAAGAACA; Antisense-UGUUCUUCCAGAUUCUCAGUGACUC.

### RNA isolation and qRT-PCR

The total RNA of cells was isolated according to the TRIZOL manufacturer’s instructions and further reversed transcribed to cDNA with a reverse transcription kit. The relative expression of targeted genes to GAPDH was evaluated by RT-qPCR. The primer sequences of the targeted genes are as follows: GAPDH-F: GGAGCGAGATCCC TCCAA AAT; GAPDH-R: GGCTGTTGTCATACTTCTCATGG. *FADD*-F: GTGGCT GACCT GGTACAAGAG; *FADD*-R: GGTAGATGCGTCTGAGTTCCAT.

### Cell proliferation assay

Approximately 2 × 10^4^ transfected cells with or without 5 μM 5-Fluorouracil (5-FU) were seeded in 12-well plates at 0 h. Subsequently, the total cells were detected at 24 h, 48 h, 72 h, and 96 h. Generally, the cell proliferation activity was evaluated by the cell numbers.

### Transwell assay

Approximately 5 × 10^4^ transfected cells were seeded in the transwell upper chamber with 200 µl serum-free medium, and 600 µl DMEM with 10% FBS was placed on the bottom. After 24 h, migrating cells were analyzed by staining with crystal violet. The invasion assay was done with 100 µl of 1:8 diluted Matrigel in the bottom of the upper chamber.

### Statistical analysis

Statistical analysis was carried out in SPSS software (p < 0.05*; p < 0.01**; p < 0.001***; p < 0.0001****). Student’s *t* test, one- or two-way ANOVA and Kaplan–Meier curves along with long rank tests were used in present study.

## Results

### PARGs were related to HNSCC prognosis

**Figure 1:**
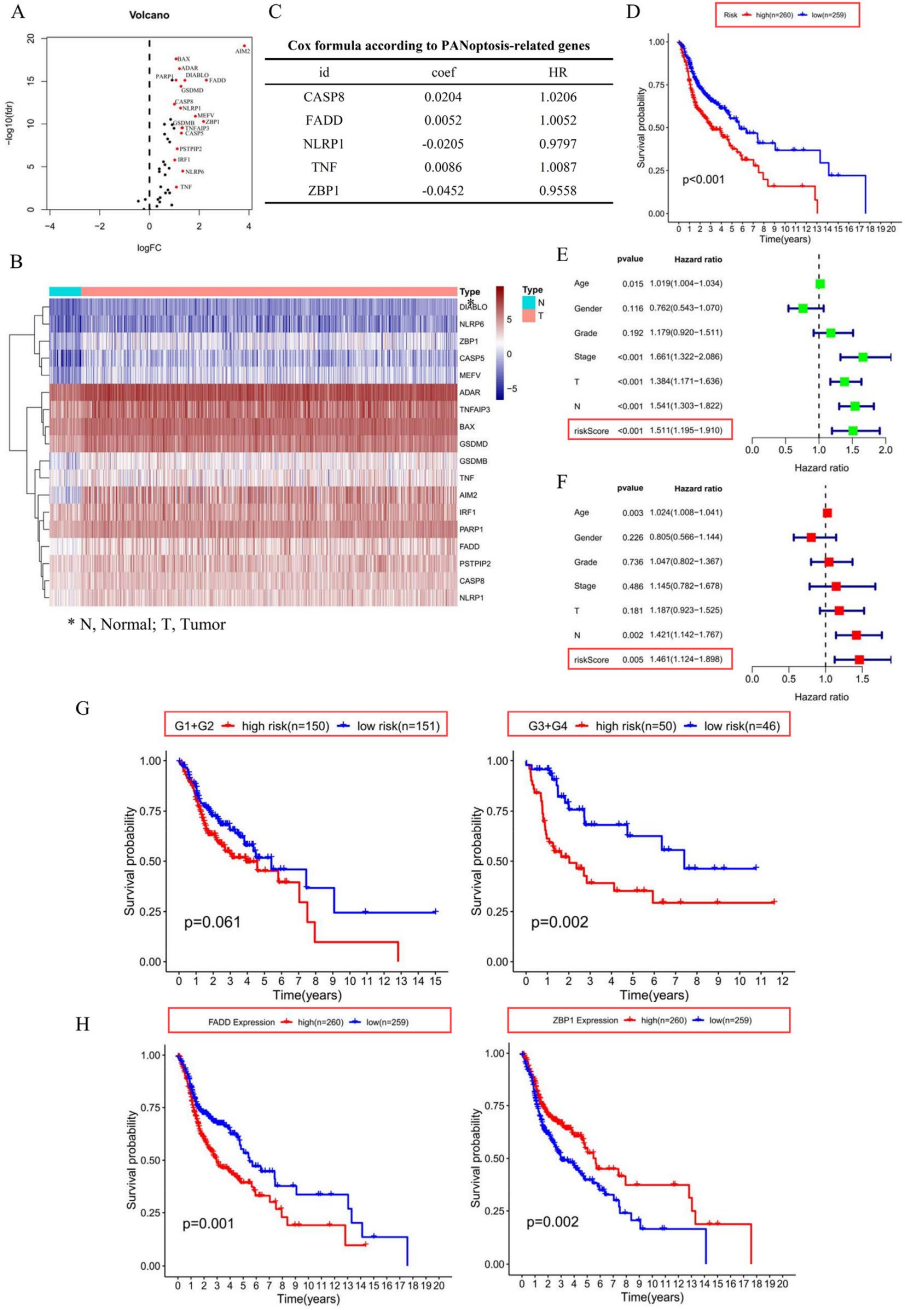
PANoptosis played a crucial role in HNSCC prognosis. (**A**,**B**) Differentially expressed PANoptosis-related genes (DEPARGs) in HNSCC were visualized by volcano and heatmap, respectively. A total of 18 PARGs were differentially expressed in HNSCC. N: normal cases; T: tumor cases; blue: downregulated genes; red: upregulated genes. (**C**) A Cox model 5 DEPARGs including *CAPS8*, *FADD*, NLRP1, TNF and *ZBP1* were built through regression analysis. (**D**) Risk score of each patient was calculated, following dividing patients into high- and low-risk group according to risk score median, which suggested that patients with high-risk score showed unfavorable overall survival. High and low means high-risk level and low-risk level, respectively. The number in the parenthesis is the size in each group. (**E**) Risk score according to DEPARGs was identified as a candidate prognostic biomarker for HNSCC by univariate Cox analysis. (**F**) Risk score based on DEPARGs was identified as an independent prognostic biomarker for HNSCC by multivariate Cox analysis. The confidence interval was given with HR. T: T classification in TNM system (tumor size); N: N classification in TNM system (lymph node metastasis); Stage: clinical stage for HNSCC samples in TCGA database; Grade: differentiation grade for HNSCC samples in TCGA database. (**G**) Survival analysis in line with stratification analysis of Grade level, suggesting that risk score might be a predictor for differentiation grade. High and low means high-risk level and low-risk level, respectively. (**H**) Survival analysis according to *FADD* and *ZBP1* expression in TCGA database. The number in the parenthesis is the size in each group.

A total of 18 DEPARGs were identified in HNSCC (Fig. 1A,B, Table 1). To further investigate the prognostic value of these genes, we conducted Cox regression analysis and constructed a risk model including *CASP8*, *FADD*, *NLRP1*, *TNF*, and *ZBP1* (Fig. 1C), suggesting that patients with high-risk levels had lower overall survival rate (Fig. 1D). Moreover, univariate Cox analysis indicated that the risk score according to PARGs might be a candidate prognostic factor for HNSCC (Fig. 1E). Furthermore, risk score was identified as an independent biomarker by multivariate Cox regression analysis (Fig. 1F). In addition, survival analysis combined with grade level could also predict HNSCC prognosis (Fig. 1G). Subsequently, we determined the prognostic value of variables in the risk model, and found that Z-DNA binding protein 1 (*ZBP1*) and *FADD* were candidate prognostic biomarkers for HNSCC (Fig. 1H).

**Table 1:** Differentially expressed PANoptosis-related genes in HNSCC.

Gene	log_2_FC	p-value	FDR
*ADAR*	1.2075	< 0.0001	< 0.0001
*AIM2*	3.8181	< 0.0001	< 0.0001
*BAX*	1.0694	< 0.0001	< 0.0001
*CASP5*	1.2851	< 0.0001	< 0.0001
*CASP8*	1.0060	< 0.0001	< 0.0001
*DIABLO*	1.4231	< 0.0001	< 0.0001
*FADD*	2.2858	< 0.0001	< 0.0001
*GSDMB*	1.3066	< 0.0001	< 0.0001
*GSDMD*	1.2674	< 0.0001	< 0.0001
*IRF1*	1.0160	< 0.0001	< 0.0001
*MEFV*	1.8459	< 0.0001	< 0.0001
*NLRP1*	1.2504	< 0.0001	< 0.0001
*NLRP6*	1.3386	< 0.0001	< 0.0001
*PARP1*	1.0693	< 0.0001	< 0.0001
*PSTPIP2*	1.1166	< 0.0001	< 0.0001
*TNF*	1.0830	0.0017	0.0023
*TNFAIP3*	1.3259	< 0.0001	< 0.0001
*ZBP1*	2.1735	< 0.0001	< 0.0001

We further investigated the protein expression of *ZBP1* and *FADD* in the Human Protein Atlas (HPA) database. *FADD* protein level was overexpressed (Fig. S1A), while *ZBP1* protein level was nearly not expressed in tumor tissues (Fig. S1B). According to the mRNA expression level in TCGA, *FADD* was related to lymph node metastasis, and *ZBP1* might play a crucial role in tumor size as well as clinical stage (Fig. S1C). The risk score in line with PARGs was also regarded as an unfavorable indicator for lymph node metastasis and advanced stage (Fig. S1D). Taken together, these results showed that PARGs might play a vital role in HNSCC progression and prognosis.

### PARGs were related to immune infiltration of HNSCC

**Figure 2:**
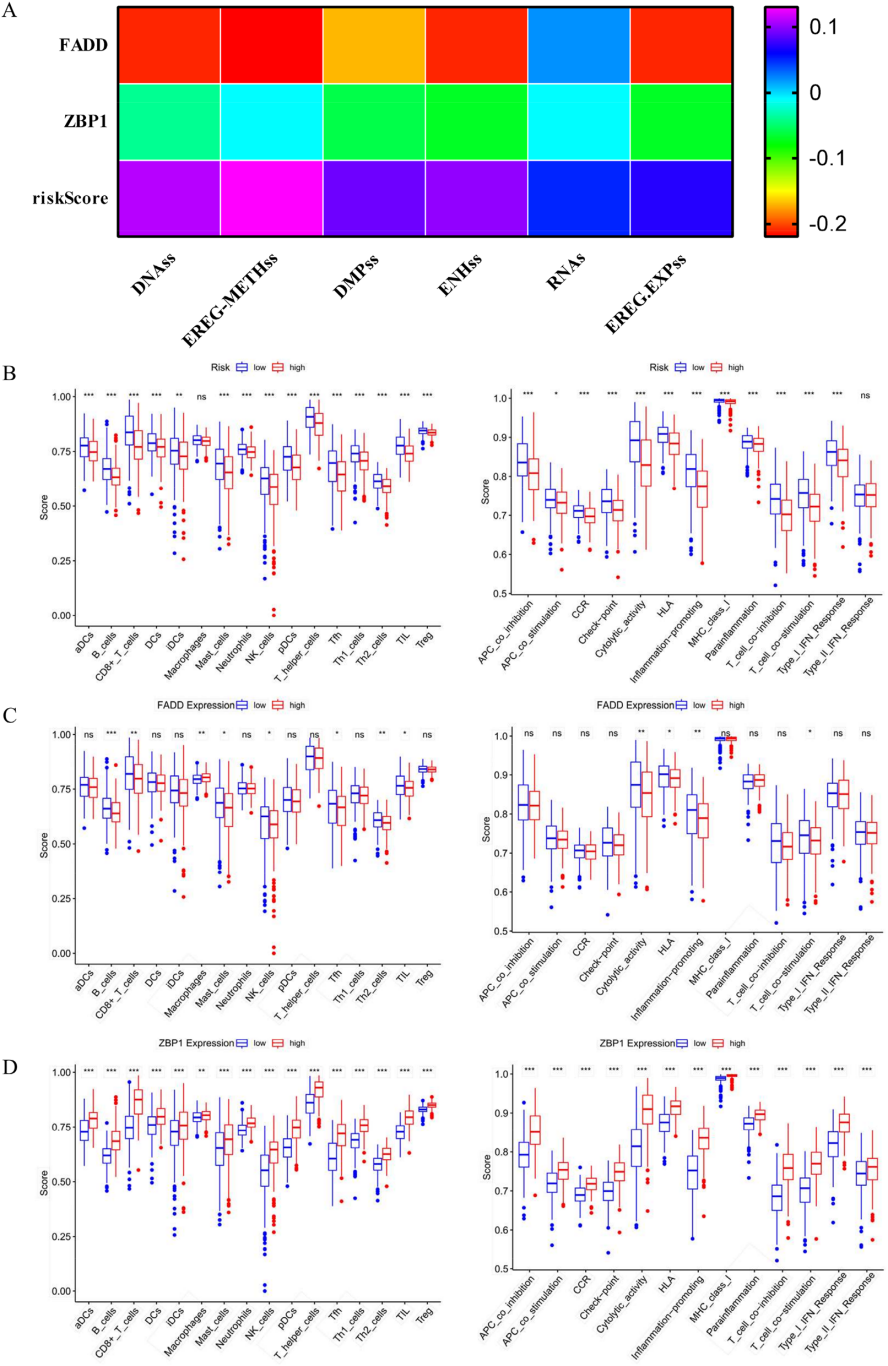
PANoptosis related to immune infiltration of HNSCC. (**A**) Heatmap showing the relationship between risk score and cancer cell stemness score according to DNA methylation (DNAss) and RNA (RNAss) in HNSCC. (**B**) Box diagram displaying the correlation between risk score and immune cell scores, and between risk score and immune function scores in HNSCC. (**C**) Box diagram displaying the correlation between *FADD* expression and immune cell scores, and between *FADD* expression and immune function scores in HNSCC. (**D**) Difference analysis between *ZBP1* expression and immune cell scores, and between *ZBP1* expression and immune function scores in HNSCC.

To further explore the role of PANoptosis in HNSCC, we analyzed the correlation between prognostic PARGs and HNSCC TME. We showed that the risk score according to the PARGs expression level was positively related to the stemness score (Fig. 2A), indicating that PARGs might play a critical role in the initiation of HNSCC. In addition, patients with high-risk scores tended to have lower immune cell scores, particularly NK cells and TILs (Fig. 2B, Table S2). Meanwhile, the immune response in high-risk level group was inhibited, in particular Type-I-IFN-response (Fig. 2B). *FADD* was identified to play a vital role in NK cell as well as TIL scores. Meanwhile, high expression of *FADD* might inhibit cell Cytolytic-activity (Fig. 2C). Although *ZBP1* was overexpressed in tumor tissues, high expression of *ZBP1* was positively related to the immune response (Fig. 2D). These results showed that PARGs were related to the immune infiltration of HNSCC.

### *ZBP1* enhanced the immunotherapy response of HNSCC

**Figure 3:**
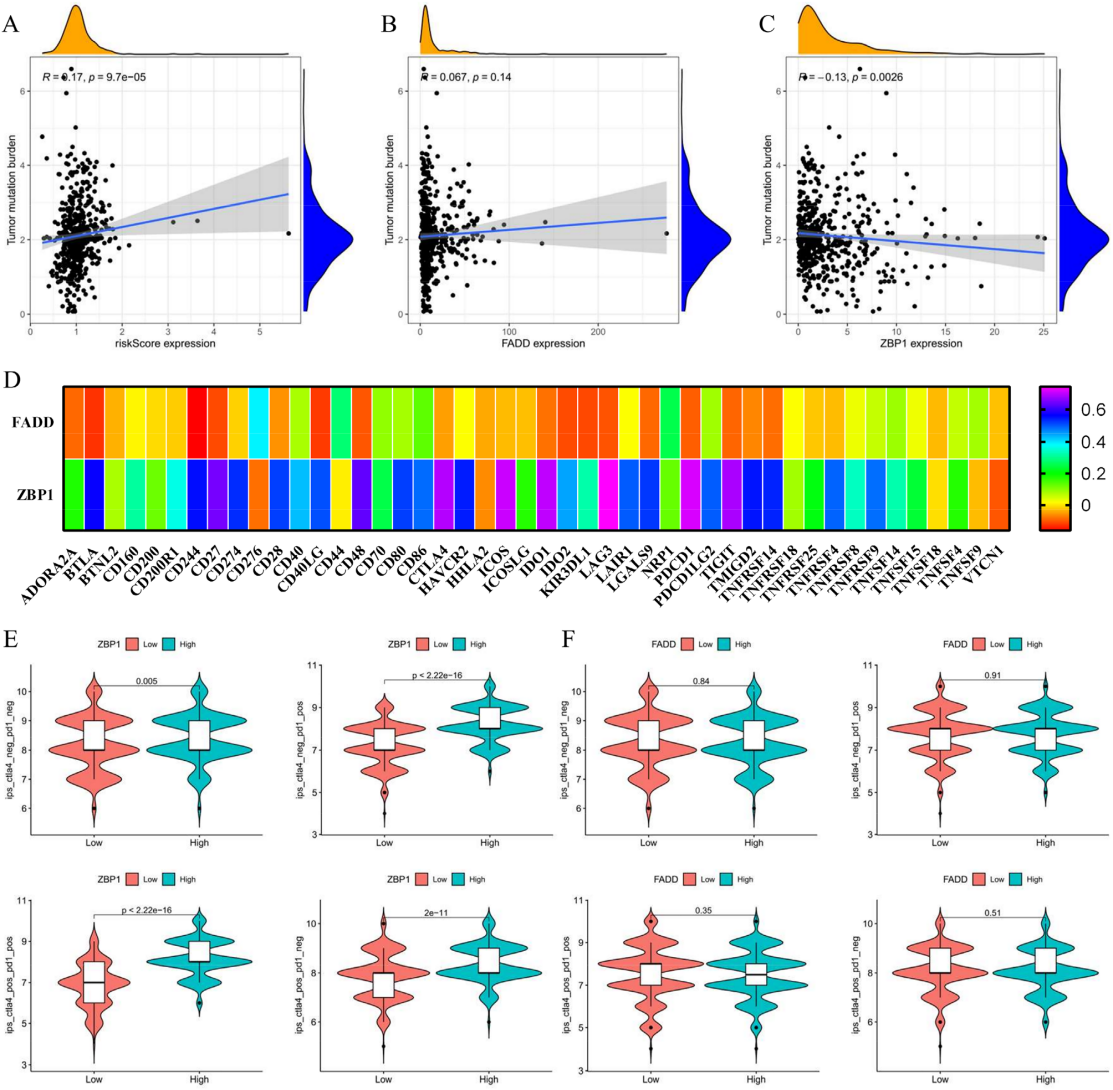
*ZBP1* enhanced immunotherapy response. (**A**–**C**) Correlation analysis between risk score according to DEPARGs, *FADD, ZBP1* and TMB, respectively. (**D**) Co-expression analysis of *FADD*, *ZBP1* expression and immune checkpoints expression. (**E**,**F**) Violin plots displaying the effect of *ZBP1* and *FADD* on the immunotherapy response of HNSCC patients. The immune cell proportion score (IPS) concerning immunotherapy data including ctla4-negative-pd1-neg, ctla4-negative-pd1-pos, ctla4-pos1-neg, and ctla4-pos1-pd1-pos were obtained from TCIA database. High expression of *ZBP1* showed better immunotherapy response, while *FADD* had no effect on HNSCC immunotherapy on basis of TCIA database.

We further investigated the role of PARGs in HNSCC immunotherapy. The risk score was negatively associated with TMB, an indicator of immunotherapy response (Fig. 3A), suggesting that patients with high-risk score had an unsatisfactory immunotherapy response. The prognostic biomarker *FADD* was also positively, while *ZBP1* was negatively related to TMB level (Fig. 3B,C), indicating that PANoptosis might play a crucial role in immunotherapy resistance. Furthermore, we showed that the expression of *FADD* and *ZBP1* was correlated with majority of immune checkpoints (Fig. 3D). Moreover, high *ZBP1* expression showed better response to immunotherapy (Fig. 3E), while *FADD* had no effect on HNSCC immunotherapy (Fig. 3F).

### PARGs were related to drug therapy

**Figure 4:**
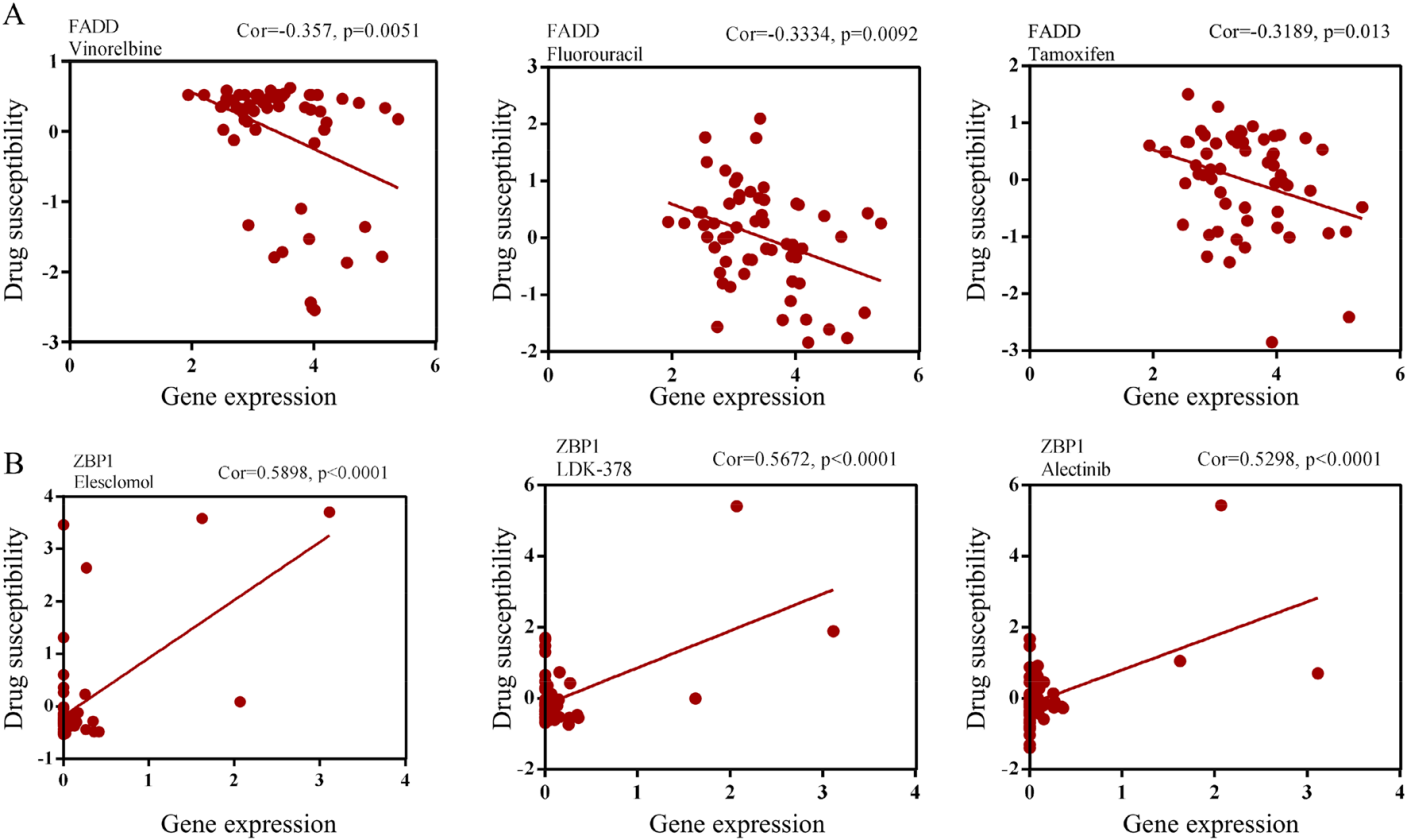
*FADD* and *ZBP1* were related to drug therapy response. (**A**) Scatter plots revealing the role of *FADD* in chemotherapy resistance. (**B**) Scatter plots showing the correlation between *ZBP1* and drug therapy susceptibility. The top 3 drugs were listed.

To explore the role of PARGs in HNSCC drug therapy, we performed a correlation analysis between drug susceptibility and prognostic biomarkers. Patients with high expression of *FADD* tended to develop chemotherapy resistance, such as resistance to fluorouracil (Fig. 4A, Table 2). *ZBP1* enhanced the response to drug therapy (Fig. 4B, Table 3). These results suggested that PANoptosis might play a significant role in the response of HNSCC to chemotherapy.

**Table 2:** Correlation between *FADD* expression and drug susceptibility.

Drug	Cor	p-value
Vinorelbine	− 0.3570	0.0051
Fluorouracil	− 0.3334	0.0092
Tamoxifen	− 0.3189	0.0130
7-Tert-butyldimethylsilyl-10-hydroxycamptothecin	− 0.2929	0.0232
By-Product of CUDC-305	− 0.2818	0.0292
Denileukin Diftitox Ontak	− 0.2729	0.0349

**Table 3:** Correlation between *ZBP1* expression and drug susceptibility.

Drug	Cor	p-value
Elesclomol	0.5899	0.0000
LDK-378	0.5672	0.0000
Alectinib	0.5298	0.0000
SR16157	0.4420	0.0004
AP-26113	0.4125	0.0011
Entinostat	0.3619	0.0045
Estramustine	0.3571	0.0051
Denileukin Diftitox Ontak	0.3477	0.0065
Itraconazole	0.3399	0.0079
Tegafur	0.3397	0.0079
Dolastatin 10	0.3233	0.0118
Ethinyl estradiol	0.3114	0.0154
Oxaliplatin	0.3113	0.0155
Isotretinoin	0.3105	0.0158
7-Hydroxystaurosporine	0.3067	0.0172
Dromostanolone propionate	0.3045	0.0180
Belinostat	0.2859	0.0268
Ixabepilone	0.2820	0.0290
Imiquimod	0.2812	0.0295
Tamoxifen	0.2794	0.0306
Palbociclib	0.2702	0.0368
Raloxifene	0.2676	0.0387
Fluphenazine	0.2641	0.0414
Eribulin mesilate	0.2571	0.0474

### *FADD* and *ZBP1* contributed to dysregulation of HNSCC-related pathways

**Figure5:**
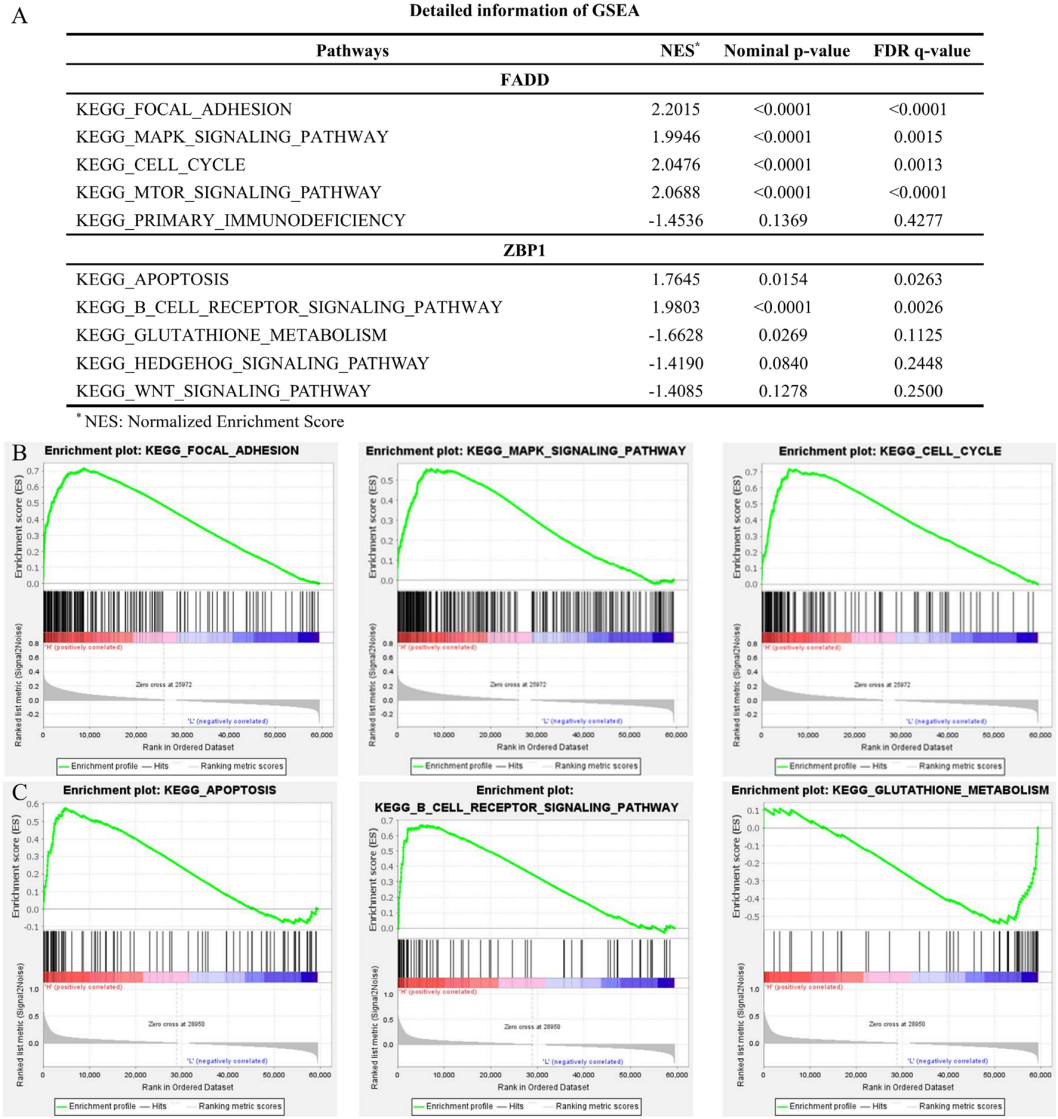
*FADD* and *ZBP1* contributed to dysregulation of various cancer-associated signaling pathways. (**A**) The detailed information of GSEA results. (**B**) GSEA suggesting the role of *FADD* overexpression in various cancer-related pathways, such as focal adhesion, MAPK signaling pathways and cell cycle. (**C**) The correlation between *ZBP1* expression and various KEGG pathways activities including apoptosis, immune response and Glutathione metabolism.

To predict the potential mechanism affected by PANoptosis, we further conducted GSEA to screen KEGG pathways influenced by *FADD* and *ZBP1* (Fig. 5A). *FADD* overexpression was associated with the activation of several cancer-related pathways, such as the MAPK and MTOR signaling pathways. Meanwhile, *FADD* contributed to cell cycle, focal adhesion and inhibition of immune response (Fig. 5B, Fig. S2A), suggesting the vital role of *FADD* in HNSCC progression. *ZBP1* might promote apoptosis and immune process. Upregulation of *ZBP1* impaired the activity of cancer-related pathways, such as Hedgehog and WNT signaling pathways (Fig. 5C, Fig. S2B). These results suggested that *FADD* and *ZBP1* might be the crucial regulators in these HNSCC-related pathways.

### Silence of *FADD* inhibited HNSCC proliferation, migration, invasion and enhance susceptibility to 5-FU

**Figure 6:**
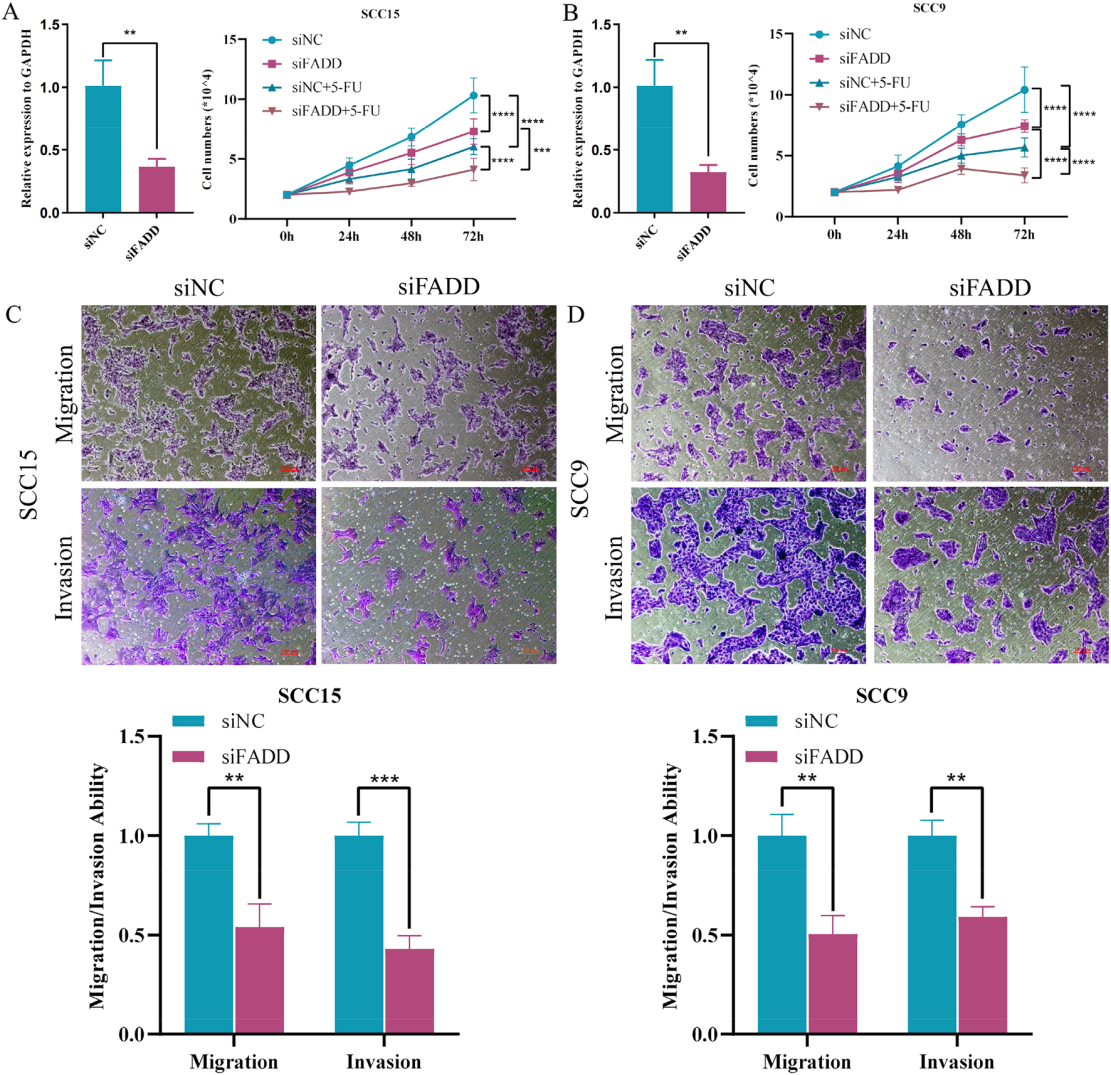
Knockdown of *FADD* inhibited the progression and enhanced susceptibility to 5-FU of HNSCC cell. (**A**,**B**) Line diagram displaying the role of FADD knockdown in HNSCC cells proliferation and susceptibility to 5-FU. Student’s T test (SCC15 p = 0.0059; SCC9 p = 0.0048) and Two-way ANOVA (SCC15 siNC vs. si*FADD* p < 0.0001, siNC vs. siNC + 5-FU p < 0.0001, si*FADD* vs. si*FADD* + 5-FU p < 0.0001, siNC + 5-FU vs. si*FADD* + 5-FU p = 0.001; SCC9 siNC vs. si*FADD* p < 0.0001, siNC vs. siNC + 5-FU p < 0.0001, si*FADD* vs. si*FADD* + 5-FU p < 0.0001, siNC + 5-FU vs. si*FADD* + 5-FU p < 0.0001) were used to statistically test the efficiency of *FADD* knockdown and cell proliferation assays, respectively. (**C**,**D**) Transwell assay showing the contribution of *FADD* in HNSCC cell migration and invasion ability after downregulation of *FADD*. (SCC15 Migration p = 0.0037, Invasion p = 0.0005; SCC9 Migration p = 0.0015, Invasion p = 0.0037). Student’s *T* test was used to conduct statistical tests for migration and invasion ability.

We further explored the biological function of prognostic biomarkers. We found that *FADD* was overexpressed in HNSCC cell lines (Fig. S3), while *ZBP1* was not detected (Data not shown). Furthermore, we showed that knockdown of *FADD* inhibited HNSCC cell proliferation, and inhibition of *FADD* might elevate the susceptibility of HNSCC cells to 5-FU (Fig. 6A,B). Moreover, silencing *FADD* impaired the migration and invasion abilities of cancer cells (Fig. 6C,D). These results suggested that *FADD* might promote HNSCC progression and showed promise as a candidate therapeutic target to enhance chemotherapy.

## Discussion

As a newly identified PCD mode, PANoptosis contributes to anti-tumor immunity by regulating the enrichment of immune cells and then promoting the death of cancer cells^20^. Aberrant PANoptosis status played a crucial role in the therapy and prognosis of various malignancies. In esophageal cancer, PANoptosis mediated by sulconazole could induce oxidative stress and impair aerobic glycolysis to enhance radiosensitivity^21^. Xiong et al. identified that PANoptosis might be an important indicator for efficacy of immunotherapy and chemotherapy in hepatocellular carcinoma (HCC)^22^. Wei et al. also showed that PANoptosis was associated with HCC overall survival, immune infiltration, and immunotherapy response^23^, suggesting that PANoptosis showed promise to be a therapeutic target. The dysregulation of cell proliferation and death plays a crucial role in the tumorigenesis of HNSCC, which is characterized by therapy resistance and infiltration of immune cells, contributing to unfavorable prognosis^24,25^. Currently, the therapy response and immune status of HNSCC patients have been revealed in accordance with various forms of gene sets related to different forms of cell death^26^. For instance, Huang et al. suggested that a signature based on ferroptosis-associated gene showed great predictive power as an independent indicator of prognosis, and patients at low-risk may benefit from immunotherapy^26^. Recently, Gao et al. demonstrated the effectiveness of PANoptosis associated molecular clustering and prognostic features in predicting the prognosis and immune landscape of HNSCC^13^. Therefore, targeting PANoptosis might enhance the progress of more effective treatment strategies for HNSCC immune- and chemotherapy. A better understanding of the role of PANoptosis in HNSCC might represent an effective therapeutic target for HNSCC in clinics.

In this study, 18 PARGs were identified as differentially expressed genes in HNSCC, and five genes, including *FADD*, *CASP8*, *NLRP1*, *TNF,* and *ZBP1*, were enrolled in the prognostic model. The risk-score might be regarded as an independent biomarker for predicting the prognosis of HNSCC by univariate and multivariate Cox regression analysis, as well as a predictor of lymph node metastasis and advanced clinical stage. Risk-score was positively related to TMB level, which suggested that these five genes might be candidate immunotherapeutic targets for HNSCC. Moreover, we showed that risk score was negatively related to 14 immune cell scores, especially B cells, T cells, NK cells and TILs. Meanwhile, the dysregulation of PANoptosis might inhibit the immune process. Taken together, PANoptosis might play a crucial role in the prognosis and immune infiltration of HNSCC. We further found that *FADD* and *ZBP1* were associated with the prognosis of HNSCC patients, and contributed to immune infiltration. *ZBP1*, as an innate sensor, was associated with inflammasome activation, inflammation, and cell death^27^. Recently, *ZBP1* was identified to act as a crucial regulator in multiple PCD pathways^28,29^. Rajendra Karki et al. demonstrated that downregulation of *ZBP1* could promote tumorigenesis by suppressing PANoptosis and impairing immune response^30^. In addition, *ZBP1* plays a role in the immune response via mediating type-I interferon production^27,31^, suggesting that *ZBP1* is fundamental to informing therapeutic strategy. Fu et al. demonstrated the critical role of *ZBP1* in inducing tumor-associated proteins^32^. Upregulation of *ZBP1* could enhance PANoptosis via inducing TNF and IFN-γ and then inhibit tumorigenesis^33^, while the immune response and cell death were impaired when *ZBP1* was downregulated^30^. On the other hand, inhibition of *ZBP1* expression might increase the migration of ovarian cancer, and impair fisetin-induced apoptosis^34^. Deletion of *ZBP1* blocked tumor necroptosis during tumor development and inhibited breast cancer metastasis, suggesting that *ZBP1* is the key regulator of tumor necroptosis and provides a potential therapeutic target for controlling tumor metastasis^35^. In HNSCC, we suggested that upregulation of *ZBP1* enhanced the immunotherapy response of HNSCC patients in the TCGA database. However, *ZBP1* protein expression was nearly not detected on basis of HPA database. Possibly, there are various post-transcriptional regulation of *ZBP1* in HNSCC. Currently, *ZBP1* could interact with various mRNAs, and play a key role in the post-transcriptional regulation of gene expression^36^. Generally, mRNAs could be directly interacted with the KH34 domain of *ZBP1*, leading to post-transcription and then regulating their corresponding protein expression^37^. Numerous studies have focused on the role of *ZBP1* in regulating post-transcription. Nevertheless, fewer studies have discussed the post-transcription of *ZBP1*. Hence, elaborating on the post-transcription of *ZBP1* might help reveal the potential mechanism of HNSCC tumorigenesis and progression. As an adaptor molecule of PANoptosis and apoptosis, *FADD* can interact with various cell surface receptors and mediate cell apoptotic signals. In addition, *FADD* could be recruited by various molecules through its C-terminal death domain, such as TNFRSF6/Fas-receptor, tumor necrosis factor receptor, TNFRSF25, thereby participating in the death signaling initiated by these receptors^38^. Moreover, *FADD* can regulate various signaling pathways related to cell death, such as caspase activation via the extrinsic apoptotic signaling pathway, ligand-dependent caspase activation and regulation of necroptotic cell death^39,40^. In addition, *FADD* could prevent the spontaneous expression of *ZBP1*, and then inhibit necroptosis of cancer cells^41^. Dysregulation of *FADD* contributes to the tumorigenesis and progression of various cancers^42^. For instance, *FADD* protected cancer cells from drug-induced apoptosis in pancreatic cancer, while impairment of *FADD* expression sensitized drug-resistant cells to Adriamycin^®^-mediated apoptosis^43^ suggesting that *FADD* was a crucial chemotherapeutic target. In penile squamous cell carcinoma (PSCC), overexpression of *FADD* was an adjunct biomarker with poor prognosis in PSCC, and might be regarded as a tumor immune environment regulator^44^. In lung cancer, Wei et al. suggested that *FADD* is one of the prominent risk factors. Inhibition of *FADD* could reduce cancer cell proliferation, and knockdown of *FADD* elevated the apoptosis and pyroptosis of cancer cells^45^. We also showed that *FADD* contributed to unfavorable overall survival and immune infiltration of HNSCC, suggesting that *FADD* might be a crucial oncogene in squamous cell carcinoma. Studies showed that amplification of 11q13.3 is related to increased metastasis in HNSCC^46^, while *FADD* was considered to be a driver gene in amplification of the chromosomal 11q13.3 region, thereby contributing to oncogene expression, such as *cyclin D1*^47^. Pattje WJ et al. suggested that *FADD* was related to regional and distant metastases, and might be visualized as a therapy target to reduce the risk of distant metastases^48^. A recent Meta-Analysis also identified the crucial role of *FADD* in the prognosis of HNSCC^49^. We found that *FADD* was related to poor overall survival, and enhanced chemotherapy resistance. Wei et al. also suggested that *FADD* as the critical regulator of PANoptosis was related to therapy resistance in HCC^23^. On the other hand, we identified that high *FADD* expression might inhibit the immune process via regulating NK cells and TIL, which contributed to immune escape of cancer cells. Meanwhile, we conducted biological experiments to verify that *FADD* enhanced the proliferation, migration and invasion of HNSCC cancer cells, indicating that *FADD* showed promising diagnostic and prognostic significance in HNSCC. In conclusion, these results demonstrated that *FADD* was expected to be a therapeutic target.

In summary, we conducted a PANoptosis-based molecular signature, and identified that PANoptosis played a crucial role in predicting prognosis, TMB, and guiding drug and immunotherapy. Moreover, we suggested that *FADD* might be a potential therapeutic target for HNSCC. These findings in this study might enhance the understanding of PANoptosis and help to screen more effective treatment strategies for HNSCC.

### Limitations

Our study may have some vital clinical significance, but there are some limitations. Firstly, we fail to investigate the mechanism of *FADD*-mediated PANoptosis in HNSCC. Then, the role of *FADD* in HNSCC should be explored in vivo. Thirdly, the roles of *FADD* in immune escape and therapy resistance need to be determined. Finally, the post-transcriptional regulation and the role of *ZBP1* overexpression in HNSCC also need to be further explored.

### Supplementary Information

Supplementary Information.

## Data Availability

All the data in this study were obtained from public database. All of the data presented in this study could be obtained from corresponding author.
