# Supplementary material for: Identification of PANoptosis-related biomarkers and analysis of prognostic values in head and neck squamous cell carcinoma
Source: Sci Rep. 2024 Apr 29;14:9824. doi: 10.1038/s41598-024-60441-8 (PMC11058810; doi:10.1038/s41598-024-60441-8)
Supplement: Supplementary file 1 — Supplementary Information. [file 41598_2024_60441_MOESM1_ESM.doc]

**FigureS1**

**
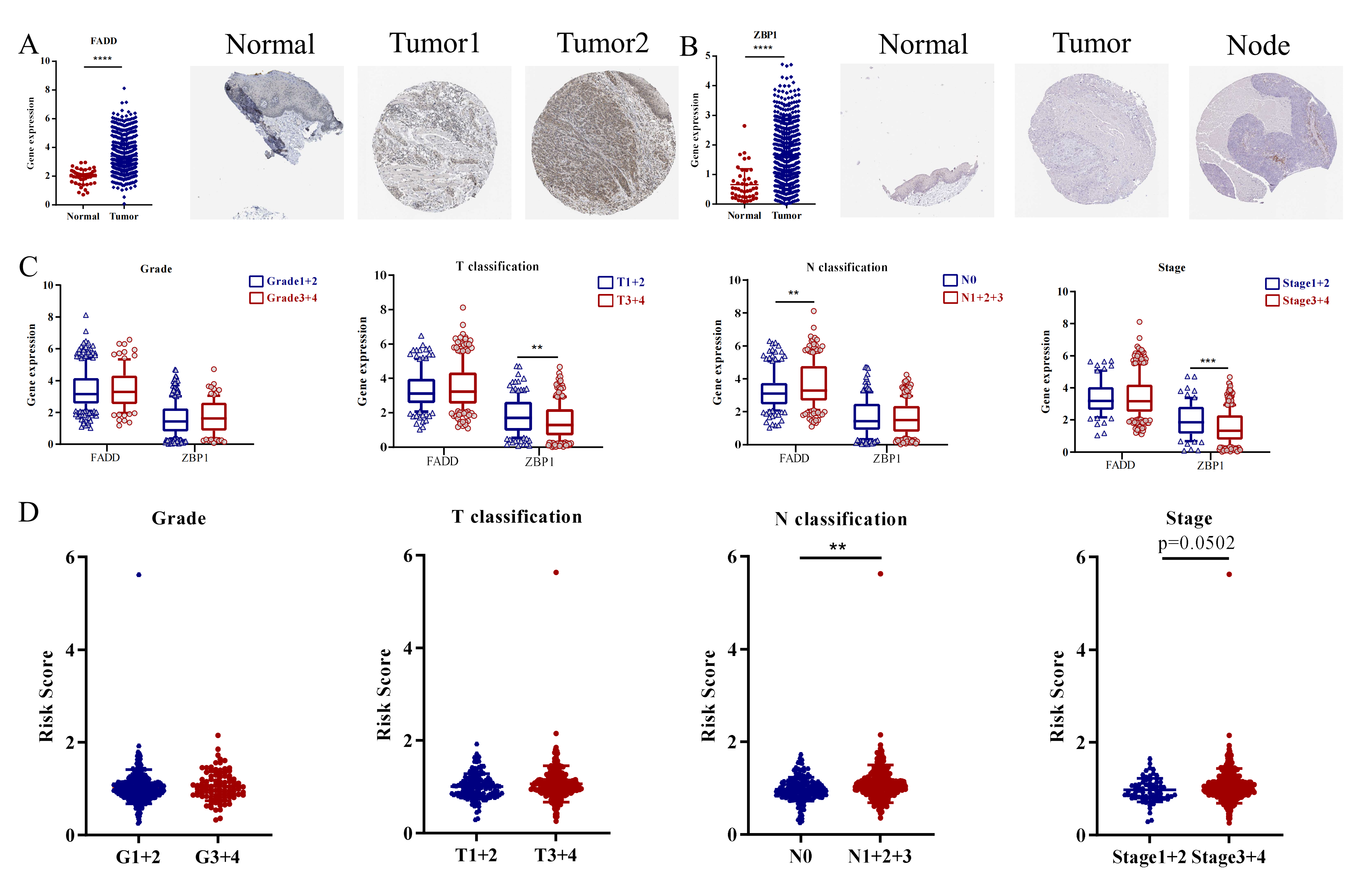
**

**Figure S1 Correlation of Clinical characteristics.** A; B, Scatter diagram and IHC images showing the *FADD* and *ZBP1* expression in HNSCC cases, respectively. C, Differential *FADD* and *ZBP1* expression in differentiation grade, T and N stage in TNM system, and clinical stage. D, Scatter diagram showing the predictive power of risk score based on PANoptosis in grade, tumor size, lymph node metastasis and clinical stage in HNSCC.

**Figure S2**


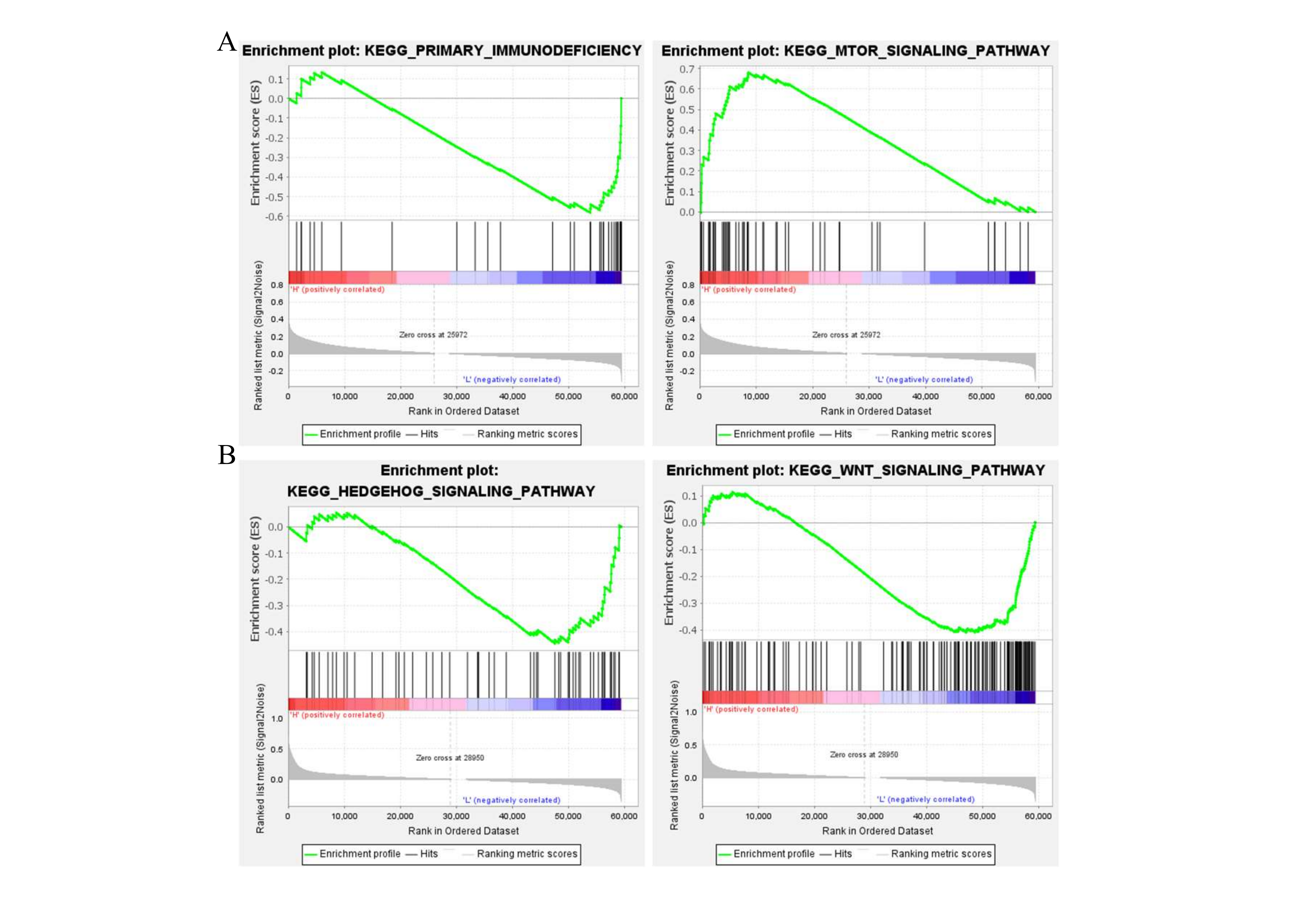


**Figure S2** A, The role of *FADD* upregulation in primary immunodeficiency and activation of MTOR signaling pathway. B, Correlation of ZBP1 expression and the activity of Hedgehog and WNT signaling pathway in HNSCC.

**FigureS3**

**
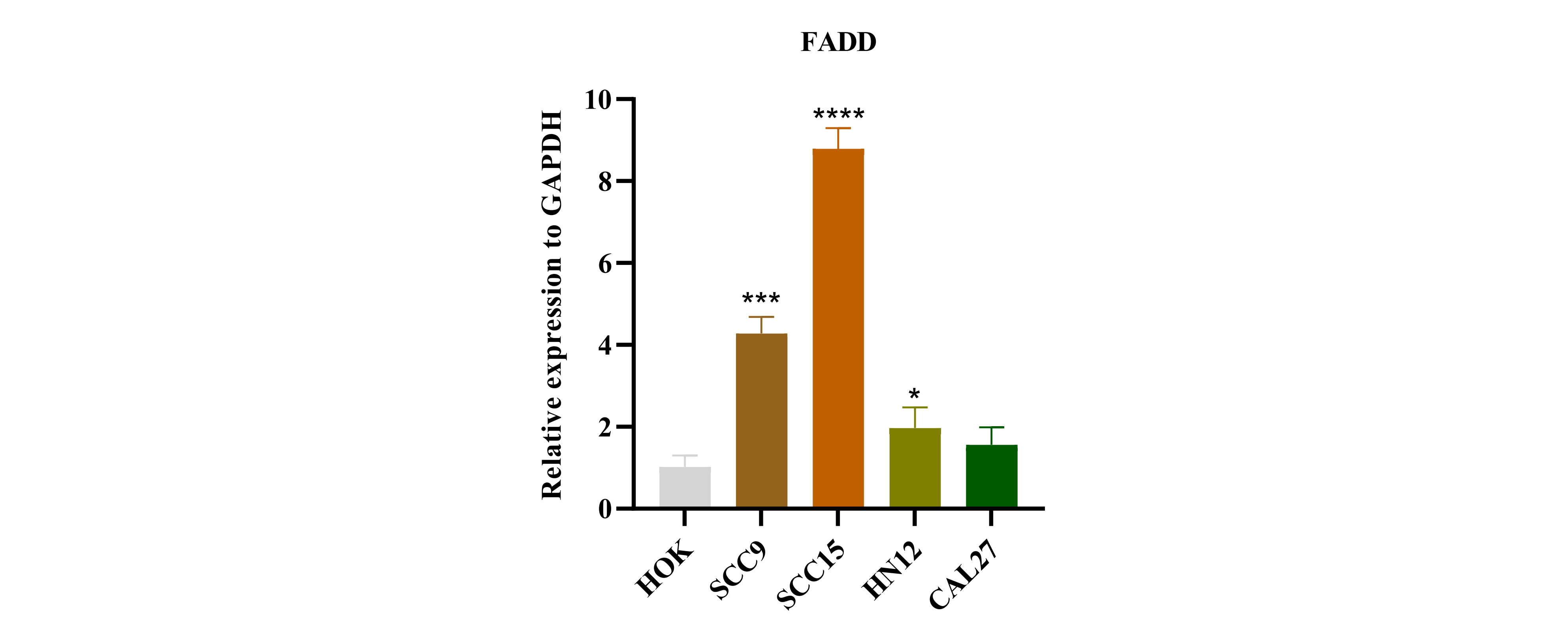
**

**Figure S3** FADD relative RNA expression level in HNSCC cell lines.

**Table S1** Identified PANoptosis-related genes in previous studies.

| **Table S1 Identified PANoptosis-related genes in previous studies** | | | |
| --- | --- | --- | --- |
| **Gene** | **Gene** | **Gene** | **Gene** |
| *ADAR* | *CASP6* | *GSDMD* | *PYCARD* |
| *AIM2* | *CASP7* | *IRF1* | *RIPK1* |
| *APAF1* | *CASP8* | *MEFV* | *RIPK3* |
| *BAK* | *CASP9* | *MLKL* | *TAB2* |
| *BAX* | *DCN* | *NAIP* | *TAB3* |
| *CASP1* | *DFNA5* | *NLRC4* | *TNF* |
| *CASP10* | *DIABLO* | *NLRP1* | *TNFAIP3* |
| *CASP12* | *FADD* | *NLRP3* | *TNFRSF1A* |
| *CASP2* | *FAS* | *NLRP6* | *TRADD* |
| *CASP3* | *GSDMA* | *NLRP9* | *ZBP1* |
| *CASP4* | *GSDMB* | *PARP1* | *NR2C2* |
| *CASP5* | *GSDMC* | *PSTPIP2* | *RBCK1* |

| **Table S2 The detailed information in Figure 2B** | | |
| --- | --- | --- |
| **Immune function score** | **Mean±SD of score**  **in low-risk level group** | **Mean±SD of score**  **in high-risk level group** |
|
| APC_co_inhibition | 0.8390±0.0556 | 0.8093±0.0583 |
| APC_co_stimulation | 0.7386±0.0378 | 0.7301±0.0397 |
| CCR | 0.7092±0.0251 | 0.6976±0.0274 |
| Check-point | 0.7336±0.0413 | 0.7107±0.0418 |
| Cytolytic_activity | 0.8815±0.0729 | 0.8320±0.0805 |
| HLA | 0.9035±0.0303 | 0.8820±0.0361 |
| Inflammation-promoting | 0.8098±0.0605 | 0.7671±0.0639 |
| MHC_class_I | 0.9920±0.0092 | 0.9895±0.0105 |
| Parainflammation | 0.8862±0.0227 | 0.8787±0.0271 |
| T_cell_co-inhibition | 0.7406±0.0581 | 0.7019±0.0588 |
| T_cell_co-stimulation | 0.7508±0.0548 | 0.7165±0.0544 |
| Type_I_IFN_Reponse | 0.8580±0.0450 | 0.8323±0.0511 |
| Type_II_IFN_Reponse | 0.7494±0.0474 | 0.7482±0.0474 |
| **Immune cell score** | **Mean±SD of score**  **in low-risk level group** | **Mean±SD of score**  **in high-risk level group** |
|
| aDCs | 0.7667±0.0610 | 0.7494±0.0582 |
| CD8+_T_cells | 0.8356±0.0926 | 0.7765±0.0904 |
| Macrophages | 0.8000±0.0286 | 0.7952±0.0295 |
| Mast_cells | 0.6787±0.1070 | 0.6425±0.1085 |
| Neutrophils | 0.7591±0.0352 | 0.7474±0.0384 |
| NK_cells | 0.6004±0.1077 | 0.5641±0.1178 |
| pDCs | 0.7180±0.0708 | 0.6761±0.0721 |
| T_helper_cells | 0.9014±0.0589 | 0.8730±0.0647 |
| Th2_cells | 0.6116±0.0441 | 0.5888±0.0472 |
| TIL | 0.7762±0.0526 | 0.7404±0.0516 |
| Treg | 0.8438±0.0189 | 0.8354±0.0180 |

**Table S2** The detailed information in Figure 2B
